# Case Report: Re-expansion pulmonary edema following a pneumothorax drainage in a patient with H1N1 and *Mycoplasma pneumoniae* co-infection

**DOI:** 10.3389/fmed.2025.1707288

**Published:** 2026-01-05

**Authors:** Haoyu Wang, Liyuan Peng, Weiwei Qian, Yarong He

**Affiliations:** 1 Emergency Department, West China Hospital, Sichuan University, Chengdu, Sichuan, China; 2 Emergency Department, Chengdu Shangjin Nanfu Hospital, Chengdu, Sichuan, China; 3 Intensive Care Unit, Affiliated Hospital of Chengdu University, Chengdu, Sichuan, China

**Keywords:** re-expansion pulmonary edema, pneumothorax, influenza A (H1N1), *Mycoplasma pneumoniae*, exacerbate lung injury

## Abstract

**Background:**

Re-expansion pulmonary edema (RPE) represents a rare but potentially fatal complication that can occur subsequent to pneumothorax drainage or pleural effusion. Currently, there is a limited understanding of its underlying pathogenesis and associated risk factors. Co-infection with *Mycoplasma pneumoniae* and influenza A (H1N1) virus, although rare, may exacerbate lung injury and complicate clinical prognoses.

**Case presentation:**

Herein, we report a case of a 20-year-old male with no prior significant medical history. The patient presented with fever and chest tightness and was subsequently diagnosed with H1N1 influenza, *Mycoplasma pneumoniae* pneumonia, and right-sided massive spontaneous pneumothorax. Despite the implementation of early closed thoracic drainage with preventive measures against RPE, the patient developed RPE and refractory pneumothorax, ultimately requiring thoracoscopic surgical intervention. Notably, invasive mechanical ventilation was not required, and the patient achieved a full recovery following intensive care management.

**Discussion:**

This case underscores the intricate pathophysiological interplay between viral and atypical bacterial co-infection. These interactions contribute to the fragility of the lung parenchyma, facilitate the development of pneumothorax, impede the healing process, and potentially elevate the risk of RPE. Notably, even in young patients who are not ventilated and have no pre-existing lung disease, severe pulmonary complications can emerge rapidly in the setting of mixed infections.

**Conclusion:**

Clinicians should remain a high level of vigilance for refractory pneumothorax and RPE (recurrent pneumothorax with empyema, assuming this is the correct expansion; if not, replace accordingly) in patients presenting with complicated pulmonary infections. Special attention should be directed toward young patients who were previously in good health and have a relatively short disease duration. Meticulous drainage strategies, close surveillance, and early contemplation of surgical intervention are of utmost importance for optimizing patient outcomes. There is a pressing need for further high-quality research to refine prevention guidelines and enhance the management of RPE in intricate clinical scenarios.

## 1 Introduction

Re-expansion pulmonary edema (RPE) is a rare but potentially fatal complication. It most frequently occurs subsequent to procedures including closed thoracic drainage, thoracentesis, and thoracic mass surgery (1). Although numerous studies have shown that the incidence of RPE following closed thoracic drainage or thoracentesis is less than 1%, respiratory failure due to RPE remains exceptionally rare, with a reported mortality rate reaching up to 20% (2–4). Due to its low incidence and the dearth of high-quality clinical investigations, research regarding the incidence, risk factors, and pathophysiology of RPE is circumscribed, and its exact mechanisms remain elusive.

Since the influenza 2009 pandemic, influenza A (H1N1) has emerged as a paramount global public health concern. It is accountable for hundreds of thousands of annual deaths (5–7). Influenza outbreaks not only impose substantial economic burdens but also pose significant threats to global health security. Secondary infections have been identified as a major determinant of mortality in patients with influenza pneumonia (8, 9). Although *Mycoplasma pneumoniae* (*M. pneumoniae*) infections have become increasingly prevalent worldwide in recent years (10–12). However, co - infection involving H1N1 and *M. pneumoniae* remains relatively infrequent, with a reported co-infection rate of only 2.94% (13). Notwithstanding this low incidence, such co - infections typically manifest with more severe clinical features and are associated with elevated mortality rates.

Notably, pneumothorax, a known complication of pulmonary infections, has not been previously reported in the context of H1N1 co-infected with *M. pneumoniae*. Herein, we report a rare case of a young adult who was first diagnosed at our hospital with H1N1 and *M. pneumoniae* pneumonia complicated by a large right-sided pneumothorax. Following closed thoracic drainage, the patient developed PRE. The refractory pneumothorax was eventually resolved through thoracic surgery. In addition to presenting this rare and complex case, we conduct a brief literature review to emphasize the significance of comprehensive evaluation, meticulous clinical decision - making, and proactive strategies for preventing fatal RPE in complex clinical scenarios.

## 2 Case presentation

A 20-year-old male, with no prior significant medical history, presented to the emergency department (ED) of our hospital at 23:51. He reported having had a fever for 1 day and experiencing chest tightness for 1 h. One day earlier, the patient had onset of fever, reaching a maximum temperature of 39 °C. This was accompanied by a series of symptoms including throat itching, nasal congestion, rhinorrhea, cough with sputum production, generalized myalgia, fatigue, dizziness, headache, and intermittent upper abdominal pain. He denied nausea, vomiting, visual or speech abnormalities, chest pain, dyspnea, diarrhea, or urinary symptoms. The patient had self-medicated with ibuprofen and traditional Chinese medicine (Pudilan), yet without any alleviation of symptoms. Approximately 1 h before presentation, the patient developed chest tightness and discomfort, which led to his visit. Since the onset of symptoms, he reported a poor appetite, while his bowel and bladder functions remained normal.

### 2.1 Physical examination

The patient’s body temperature measured 39.0 °C, heart rate was 79 beats per minute, respiratory rate was 20 breaths per minute, blood pressure was 109/61 mmHg, and oxygen saturation (SpO_2_) was 94% while breathing room air. During the examination, the patient was alert, oriented, and cooperative. The respiratory effort appeared normal, showing no pharyngeal congestion or tonsillar hypertrophy. Lung auscultation indicated asymmetrical breath sounds, with absent breath sounds over the right lung field and no audible crackles or wheezes. The cardiac examination yielded unremarkable findings, presenting a regular rhythm and no murmurs. The abdomen was soft, non-tender, without rebound tenderness or muscle guarding. There was no evidence of lower limb edema.

### 2.2 Laboratory and imaging investigations

**FIGURE 1:**
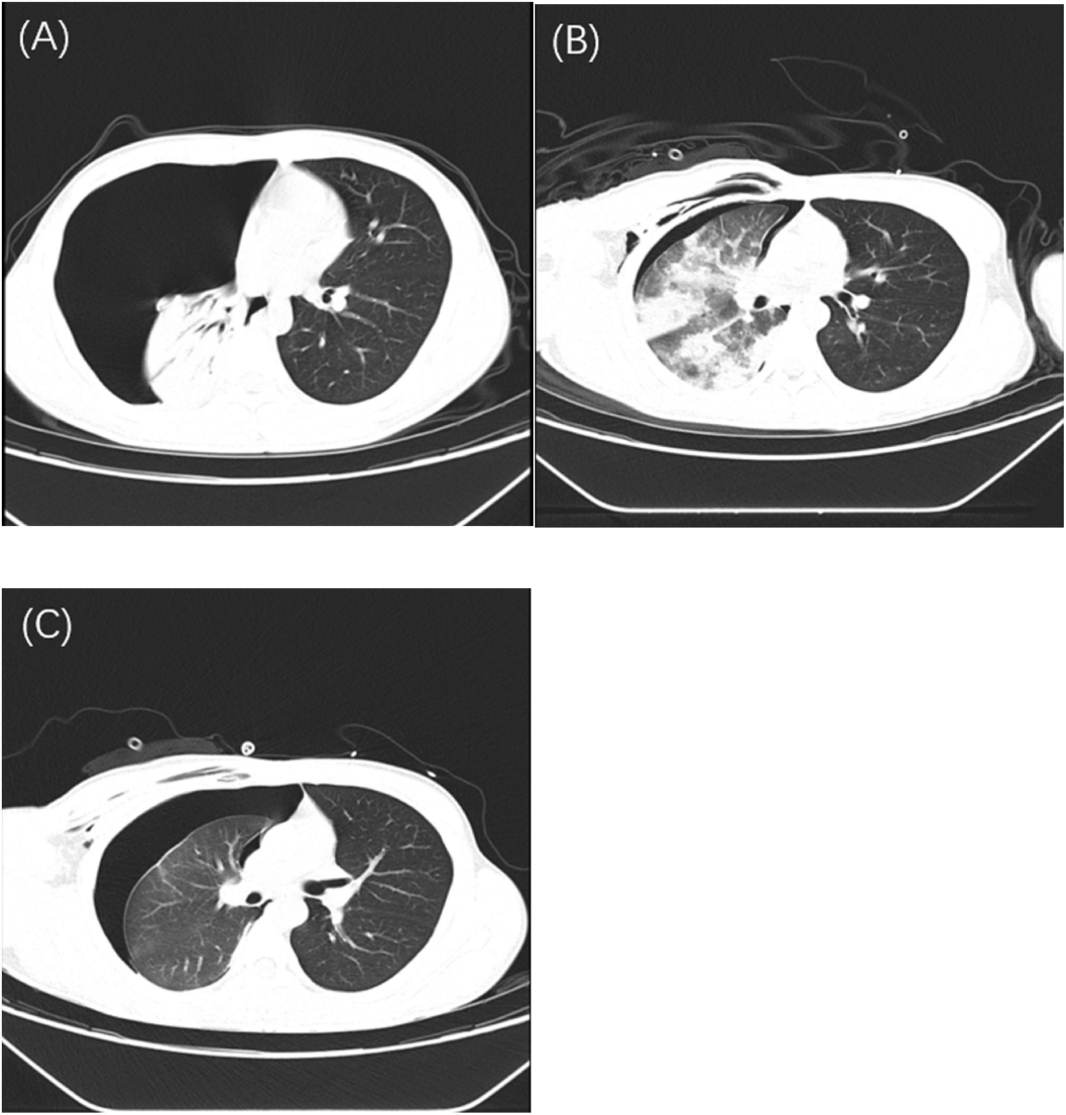
Serial chest CT images demonstrating disease progression with H1N1 and Mycoplasma pneumoniae co-infection. **(A)** Initial chest CT showing a massive right-sided pneumothorax with near-complete collapse of the right lung and minimal pleural effusion. **(B)** Chest CT performed after closed thoracic drainage, showing partial re-expansion of the right lung, persistent pneumothorax (approximately 40% compression), diffuse ground-glass opacities, patchy consolidations consistent with PRE, and subcutaneous emphysema in the right chest wall, axilla, and neck. **(C)** Follow-up chest CT after several days of continuous negative pressure drainage, demonstrating persistent massive pneumothorax, right lung atelectasis, scattered subcutaneous emphysema, and signs of pneumonia with pulmonary edema.

Chest CT revealed a large right-sided pneumothorax with a small amount of pleural effusion and right lung atelectasis (Figure 1A). No abnormalities were noted in the liver, gallbladder, pancreas, spleen, kidneys, or adrenal glands. Arterial blood gas analysis showed: pH 7.464, PaCO_2_ 32.1 mmHg, PaO_2_ 74 mmHg, base excess (BE) 0, HCO_3_^–^ 23 mmol/L, and potassium 3.6 mmol/L. Fingerstick blood glucose was 6.7 mmol/L. Laboratory tests showed hemoglobin 130 g/L, platelet count 148 × 10^9^/L, white blood cell count 4.79 × 10^9^/L, neutrophil percentage 65.3%, and monocyte percentage 13.3%. Coagulation profile and biochemistry tests were within normal limits. *Mycoplasma pneumoniae* IgM antibody was positive. Influenza A antigen test was positive. The nucleic acid test results for 13 respiratory infection-related viruses showed positive for influenza A virus, influenza A H1N1, and *Mycoplasma pneumoniae*, while adenovirus, bocavirus, rhinovirus, parainfluenza virus, Chlamydia, metapneumovirus, influenza B virus, influenza A H3N2, coronavirus, and respiratory syncytial virus tested negative. The quantitative nucleic acid test for human cytomegalovirus, real-time fluorescence detection of EB virus DNA, *Aspergillus* galactomannan test, and fungal 1,3-β-D-glucan test all returned negative results. Pre-transfusion infectious disease screening (HBV, HCV, syphilis, HIV) was negative.

The initial diagnosis included: (1) right-sided spontaneous pneumothorax; (2) atelectasis; (3) pneumonia; (4) *Mycoplasma pneumoniae* infection; and (5) Influenza A infection.

### 2.3 Clinical course and management

The patient was started on supplemental oxygen therapy. For fever management, physical cooling was combined with ibuprofen. Additionally, oral oseltamivir and intravenous levofloxacin were administered. At 02:40 on hospital day 2, bedside closed thoracic drainage was performed under local anesthesia. No intraoperative complications occurred during the procedure. After the operation, the chest tube was clamped for 20 min and then unclamped. A satisfactory water-seal fluctuation was observed.

At 03:30, the patient developed frequent coughing, respiratory distress, chest tightness, and profuse sweating, with a progressive decline in oxygen saturation. Lung auscultation revealed diminished breath sounds over the right lung and wet crackles. A diagnosis of pleural reaction and RPE was considered. The management strategy involved transitioning from nasal cannula to face mask oxygen therapy, reclamping the chest tube, administering an intravenous infusion of 5% dextrose, and administration of intravenous furosemide (20 mg) and dexamethasone (5 mg). Peripheral oxygen saturation fluctuated between 75% and 85%. Chest CT (Figure 1B) showed right-sided pneumothorax and pleural effusion with chest tube *in situ*, approximately 40% right lung compression, subcutaneous emphysema in the right chest wall, axilla, and neck, along with scattered ground-glass opacities and patchy consolidations in the right lung.

Non-invasive mechanical ventilation was initiated with the ventilation mode of Continuous Positive Airway Pressure (CPAP). Resulting in an improvement in oxygen saturation to greater than 95%. Following obtained consent from the patient and his parents, the patient was transferred to the intensive care unit (ICU) at 05:10.

In the ICU, the patient continued on non-invasive ventilation and underwent continuous negative-pressure thoracic drainage, its ventilation mode remains CPAP. On hospital day 3, he transitioned back to nasal cannula oxygen while maintaining continuous negative-pressure drainage. Repeat chest CT on hospital day 5 (Figure 1C) revealed a persistent large right-sided pneumothorax with a small amount of effusion, subcutaneous emphysema, right lung atelectasis, and signs of pneumonia and pulmonary edema.

Despite comprehensive supportive care measures, the patient still presented with persistent pneumothorax after being weaned from mechanical ventilation. Based on the joint recommendations from the thoracic surgeon and ICU physician, and due to concerns from both the patient and his family regarding the efficacy of medical management, the decision was made to proceed with surgical intervention. On hospital day 8, he underwent single-port video-assisted thoracoscopic surgery (VATS) and pleurodesis under general anesthesia.

### 2.4 Outcome and follow-up

**FIGURE 2:**
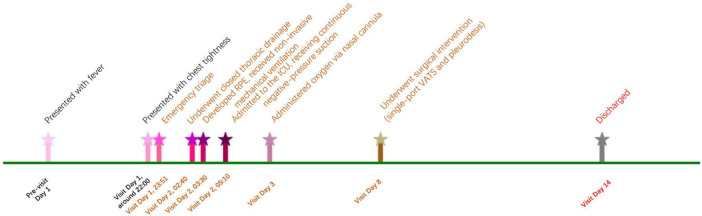
Major events and management timeline of the case.

Postoperatively, the patient had an uneventful recovery and was discharged on hospital day 14. The major events and management timeline of the case is presented in Figure 2. One-month follow-up confirmed complete recovery. Recently, we conducted a follow-up telephone call with the patient. The patient reported having a follow-up chest X-ray approximately 6 months after the surgery, the results of which were unremarkable.

## 3 Discussion

This case describes a rare and complex clinical scenario involving a previously healthy young male. Within 1 day of symptom onset, he developed spontaneous pneumothorax as a consequence of H1N1 influenza and *Mycoplasma pneumoniae* co-infection. Despite prompt diagnosis and timely closed thoracic drainage with precautionary measures aimed at minimizing the risk of RPE, the patient subsequently developed this serious complication. Notably, the pneumothorax was refractory to continuous negative-pressure drainage and ultimately required surgical intervention. Fortunately, the patient recovered without the need for invasive mechanical ventilation and was discharged without long-term sequelae. The management of this case strictly adhered to the recommendations of the British Thoracic Society Guideline for pleural disease (2023) (14). Specifically, non-invasive diagnostic and therapeutic options were given precedence; continuous negative pressure chest drainage was employed; and when clinical improvement was insufficient, timely thoracoscopic surgery with pleurodesis was carried out, among other congruent interventions.

We believe this case is unique and warrants further contemplation. The simultaneous occurrence of refractory pneumothorax and PRE in a young male with no prior health issues, who had not undergone mechanical ventilation and had a short disease course, is extremely rare in clinical practice. This is particularly true for a young patient experiencing his first episode of pneumothorax, who still required surgical intervention despite aggressive and systematic treatment. Could there be a distinct underlying pathophysiological mechanism at play? Did the co-infection with *Mycoplasma pneumoniae* and H1N1 influenza significantly influence the progression of his condition?

Co-infection with *Mycoplasma pneumoniae* and H1N1 influenza virus is uncommon, but it may have contributed to the development of pneumothorax in this patient and hindered its resolution. While both pathogens are individually known to cause pneumonia, limited research has examined their synergistic effects on lung architecture and function (13). Bacterial superinfection is a well-documented factor that exacerbates influenza severity and contributes significantly to mortality (8, 15, 16). It is therefore plausible that co-infection in this case increased lung parenchymal fragility, promoting the development of pneumothorax and complicating recovery.

The concurrent action of H1N1 and *Mycoplasma* exacerbates damage to epithelial cells and disrupts the tight junctions among cells (e.g., ZO-1), thereby leading to the impairment of the lung barrier function (17, 18). Coinfection suppresses the type I interferon (IFN) response, activates the inflammasome and associated pathways, and concurrently triggers the massive secretion of pro-inflammatory cytokines (e.g., IL-6, TNF-α, IL-1β). This forms a “cytokine storm,” causing severe immunopathological damage (18–21). H1N1 and *Mycoplasma* jointly initiate PANoptosis (panoramic programmed cell death), a complex and intense form of cell death. This process results in the release of a substantial amount of inflammatory substances and exacerbates tissue damage. For instance, it involves the rupture of the cell membrane mediated by the NINJ1 protein and the release of inflammatory factors (19, 20). The influenza virus can commandeer the host’s purine metabolism pathway to fulfill the resource demands for its rapid replication. This alteration in the metabolic environment might be exploited by *Mycoplasma* to jointly exacerbate inflammation. Examples include the activation of the purine salvage pathway mediated by purine nucleoside phosphorylase (PNP) and the activation of the APRT-AICAR-AMPK signaling axis (22).

Although pneumothorax is a recognized complication of pneumonia, it generally occurs in patients requiring mechanical ventilation or those with severe underlying disease. Its occurrence in a previously healthy, non-intubated young adult, as observed in this case, is exceptionally rare (23–25). Based on current research reports, we hypothesize that the possible mechanism is that H1N1 influenza and *Mycoplasma pneumoniae* co-infection can induce thick mucus plugging, surfactant dysfunction, and alveolar rupture through elevated intrapulmonary pressures and reduced lung compliance. This process ultimately triggers the onset of pneumothorax. As the mechanical properties of the lung deteriorate, further predisposing to complications such as refractory pneumothorax (23, 26–29).

Re-expansion pulmonary edema is a rare but serious complication that can follow rapid lung re-expansion after thoracic drainage. Its pathogenesis involves a combination of ischemia-reperfusion injury, oxidative stress, increased capillary permeability, elevated hydrostatic pressure, and impaired lymphatic clearance (30–33). Risk factors identified in previous studies include the extent and duration of lung collapse and a history of smoking (34). In this case, the patient exhibited none of the classic risk factors except for a large pneumothorax, with postoperative imaging suggesting approximately 40% lung compression. Despite gradual and controlled re-expansion via clamped drainage, RPE still occurred, underscoring the unpredictable nature of this complication, even when preventive strategies are employed.

Guidelines currently recommend limiting drainage volumes to under 1500 mL and maintaining pleural pressures above –20 cm H_2_O during thoracic interventions to mitigate RPE risk (35–38). However, these strategies are mainly applicable to pleural effusion and are difficult to implement in pneumothorax, where air volume cannot be easily quantified. Alternative techniques, such as the use of fine-bore catheters or intermittent decompression via three-way stopcocks, have been proposed to moderate re-expansion and reduce the likelihood of RPE (9, 39).

This case highlights the importance of heightened vigilance for RPE and refractory pneumothorax in patients with complex viral infections. It also underscores the need for individualized drainage strategies and enhanced monitoring protocols in high-risk cases. Future studies should focus on refining diagnostic thresholds, evaluating preventive measures, and establishing evidence-based management guidelines. Moreover, the integration of artificial intelligence and quantitative risk stratification tools may support earlier identification of susceptible patients and enable more tailored therapeutic approaches.

## 4 Conclusion

This case highlights a rare but severe instance of spontaneous pneumothorax complicated by RPE in a previously healthy young adult with H1N1 and *Mycoplasma pneumoniae* co-infection. The dual infection likely compromised lung integrity, contributing to refractory pneumothorax and RPE despite early intervention. Clinicians should remain vigilant for RPE even in low-risk, non-ventilated patients with severe or mixed infections, particular attention should be paid to young patients who were previously healthy and have a short disease course. This underscores the need for tailored drainage strategies, better preventive protocols, and further research to guide RPE management in high-risk scenarios.

## Data Availability

The raw data supporting the conclusions of this article will be made available by the authors, without undue reservation.
